# Recent advances of exosomes in immune-mediated eye diseases

**DOI:** 10.1186/s13287-019-1372-0

**Published:** 2019-08-30

**Authors:** Na Li, Lu Zhao, Yankai Wei, Vicki L. Ea, Hong Nian, Ruihua Wei

**Affiliations:** Tianjin Key Laboratory of Retinal Functions and Diseases, Eye Institute and School of Optometry, Tianjin Medical University Eye Hospital, No.251 Fukang Road, Nankai District, Tianjin, 300384 People’s Republic of China

**Keywords:** Exosomes, Sjögren’s syndrome, Corneal allograft rejection, Autoimmune uveitis, Age-related macular degeneration, Biomarkers, Drug delivery

## Abstract

Exosomes, nanosized extracellular vesicles of 30–150 nm, are shed by almost all cell types. Bearing proteins, lipids, RNAs, and DNAs, exosomes have emerged as vital biological mediators in cell-to-cell communication, affecting a plethora of physiological and pathological processes. Particularly, mounting evidence indicates that immunologically active exosomes can regulate both innate and adaptive immune responses. Herein, we review recent advances in the research of exosomes in several immune-mediated eye diseases, including Sjögren’s syndrome (SS) dry eye, corneal allograft rejection, autoimmune uveitis, and age-related macular degeneration (AMD). Additionally, we discuss the potential of exosomes as novel biomarkers and drug delivery vesicles for the diagnosis and treatment of eye diseases.

## Introduction

Exosomes were first described as 50-nm diameter-sized vesicles secreted from maturing sheep reticulocytes in the early 1980s [1, 2]. These nanovesicles sparked scientists’ interest, as they appeared to function from cellular garbage disposals to potent intercellular communication mediators. Typically, exosomes are a subtype of extracellular vesicles (EVs) (30–150 nm) secreted by almost all cell types [3, 4]. They widely exist in numerous biological fluids including serum, urine, breast milk, tear fluid, vitreous humor, saliva, and aqueous humor, under both healthy and pathological conditions [5, 6]. Encapsulated in a bilayer membrane, exosomes are enriched in various bioactive molecules, including proteins, lipids, RNAs (mRNA, circular RNA, microRNA, long noncoding RNA), and DNAs (genomic DNA, cDNA, and mitochondrial DNA) [7–9]. These molecular components are capable of inducing functional responses in recipient cells and are extraordinarily variable depending on the cellular origin and cell exposure context [10–13]. By transferring these functional molecules between cells, exosomes act as potent mediators in intercellular communication and participate in numerous physiological and pathological processes [14]. Exosomes from both immune cells and non-immune cells exert pivotal roles in the regulation of immunity [15] and have been reported to be involved in the development and treatment of inflammatory and autoimmune diseases [16, 17].

The eye, a unique sensory organ of vision, is regarded as an immune-privileged site that prevents immunogenic inflammation [18]. Still, there are several inflammatory and immune-mediated diseases which involve the anterior or posterior segment of the eye, even in severe cases resulting in sight-threatening conditions, such as Sjögren’s syndrome (SS) dry eye, corneal allograft rejection, uveitis, and age-related macular degeneration (AMD) [19–21]. Of these diseases, the action of immune cells and the expression of pro-inflammatory cytokines and chemokines induce local inflammatory responses which ultimately cause ocular tissue damage. Although therapeutic strategies have undergone substantial transformation, there are still some challenges remaining [22, 23].

In this review, we highlight and discuss the recent research advances about exosomes in several immune-related eye diseases and their potential as biomarkers and drug delivery vesicles in the eye.

## Biogenesis and function of exosomes

**Fig. 1:**
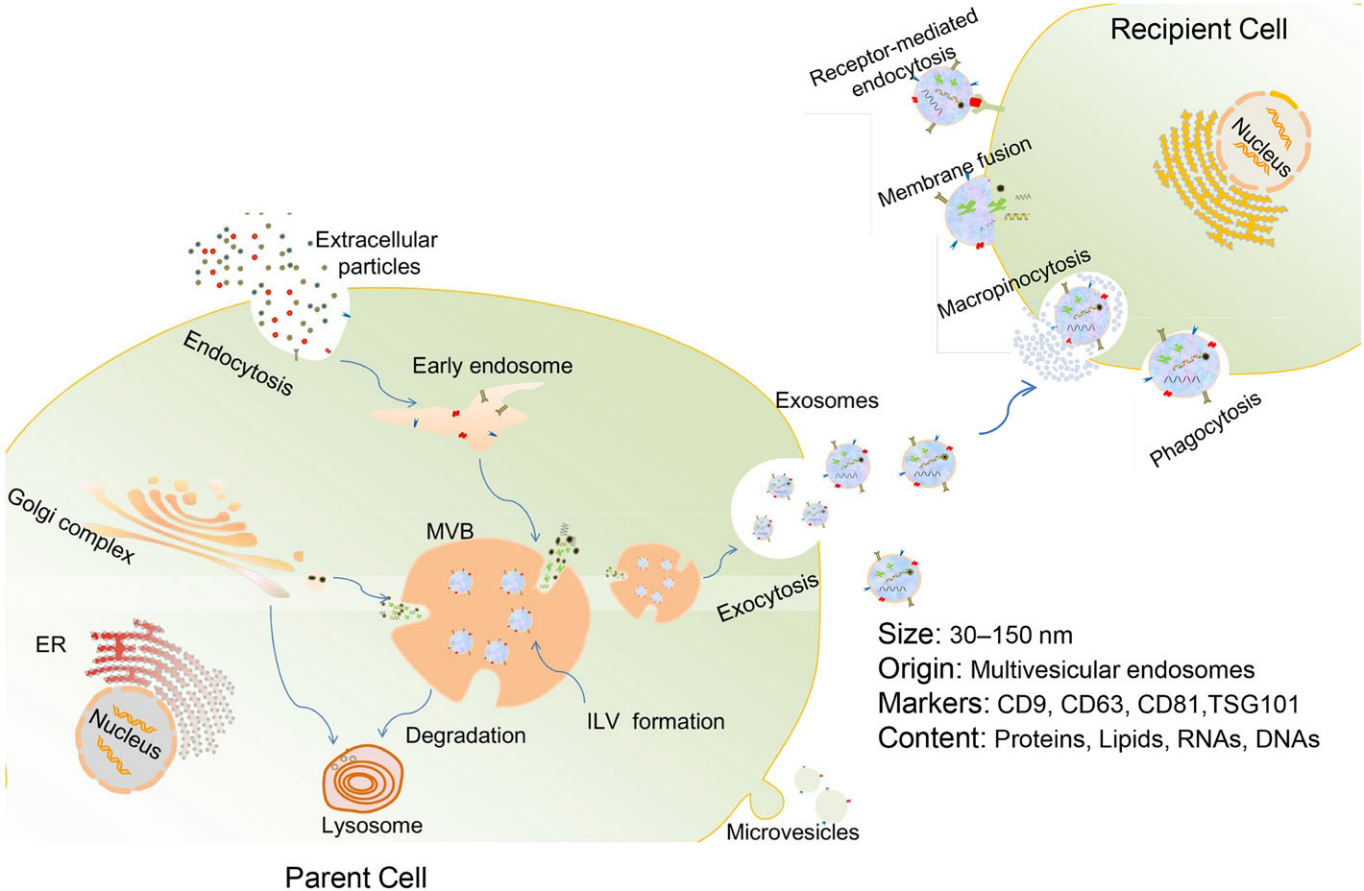
Biogenesis, release, and internalization of exosomes. Exosomes originate from early endosomes which then mature to late endosomes or MVBs. Numerous proteins, nucleic acid, and lipids are selectively encompassed in exosomes during the formation of ILV inside MVBs via the invagination of the endosomal membrane. Eventually, exosomes are released outside the cell upon fusion of MVBs with the plasma membrane. The internalization of exosomes by recipient cells can be mediated by receptor-mediated endocytosis, macropinocytosis, phagocytosis, or direct fusion of exosomes with cell membrane

Exosome generation starts with the invagination of the plasma membrane to form early endosomes. As the early endosomes mature, intraluminal vesicles (ILV) are produced in the lumen of the late endosomes (also called multivesicular bodies, or MVBs). MVBs eventually fuse with the plasma membrane and release their internal contents as exosomes. Alternatively, some MVBs are destined for degradation inside of lysosomes [3, 14] (Fig. 1). Cargoes assembled into exosomes are sorted through several molecular machinery, including the endosomal sorting complex required for transport (ESCRT) machinery (containing ESCRT-0, ESCRT-I, ESCRT-II, and ESCRT-III) and ESCRT-independent machinery (involving lipids, syndecan, and syntenin) [24, 25]. In addition, the Rab family of small GTPase proteins (such as Rab27a and Rab27b), SNARE (soluble N-ethylmaleimide-sensitive fusion attachment protein receptor) complexes, and cytoskeleton act as important modulators of exosomes secretion [24]. However, in spite of the heightened interest in this field, the mechanisms that control exosome biogenesis and secretion are still not exhaustive.

Once released, exosomes can interact with specific recipient cells. It appears that exosome targeting specificity is based on the particular combination of exosomes and acceptor cells [24]. Studies have identified that the expression of phosphatidylserine receptors, integrins, tetraspanins, lectins, glycans, and other adhesion molecules on exosome surface contributes to this process [26, 27]. Exosomes can transmit information to target cells via internalization through macropinocytosis, phagocytosis, receptor-mediated endocytosis, or membrane fusion [28–30], or via acting on their cell surface, without delivery of their cargos [31] (Fig. 1). Nevertheless, the more specific cellular and molecular basis for exosome targeting is still undetermined.

The function of exosomes was unknown until 1996, when it was found that exosomes derived from Epstein-Barr virus (EBV)-transformed B cell lines induced major histocompatibility complex (MHC) class II-restricted T cell responses in an antigen-specific manner, hinting the possible role of exosomes as mediators of immune responses [32]. Since then, intensive research has been devoted to delineating their roles in immunomodulation. It is now clearly understood that immunologically active exosomes can regulate both innate and adaptive immunity [33, 34]. Exosomes generated by immune cells have been studied extensively. For instance, exosomes from antigen-presenting cells including dendritic cells (DCs), B cells, and macrophages carry surface MHCI and MHCII molecules and thus directly stimulate CD8^+^ and CD4^+^ T cell responses, respectively [15, 35]. Besides, Okoye et al. demonstrated that Let-7d-containing exosomes derived from primary regulatory T cells (Tregs) inhibited Th1 cell responses by targeting Cox-2 in a mouse model of colitis [36]. Of note, exosome secretion in immune cells is regulated by cell context. For example, exosome release in DCs and B cells is increased after cognate T cell interactions [37–39], and mast cells produce more EVs in response to cross-linking of the high-affinity Fc receptor for IgE or exposure to calcium ionophores [40]. Aside from immune cell-derived exosomes, exosomes secreted by nonimmune cells such as tumor and mesenchymal stem cells (MSCs) have gained great attention in recent years. Tumor-cell-derived exosomes can travel to the draining lymph node, where they inhibit T cell activation by presenting programmed death-ligand 1 (PD-L1) and thus promote tumor progression [41]. Mesenchymal stem cells-derived exosomes (MSC-Exos) have been shown to enhance the differentiation of immunosuppressive cells such as M2 macrophages and Tregs, or inhibit proliferation of natural killer cells or T lymphocytes [42]. For instance, Zhao et al. discovered that mouse bone marrow-derived MSC-Exos modulated macrophage polarization by transferring miR-182, which targeted TLR4/NF-κB/PI3K/Akt signaling [43]. More attractively, several studies proposed that inflammatory stimulation increased secretion of MSC-Exos and even enhanced their anti-inflammatory and immunosuppressive properties [44–46]. However, although exosomes possess versatile biological functions including immunomodulation [47], pro-regeneration [48], anti-inflammation [49], and tumor growth regulation [50] (Table 1), the field of exosome research in eye diseases currently remains relatively less explored.

**Table 1:** A selective overview of studies reporting exosomes in diseases

Disease involved	Cellular origin of exosomes	Exosomal cargo	Biological function and (or) action mechanism	References
Colitis	Mouse Tregs	Let-7d	Suppress Th1 cell proliferation and secretion of IFN-γ	[36]
Cancer	Cancer cell lines	PD-L1	Suppress T cell activity in the draining lymph node by presenting PD-L1	[41]
Myocardial Ischemia Reperfusion	Mouse bone marrow-derived MSCs	miR-182	Modulate macrophage polarization via targeting the TLR4/NF-B/PI3K/Akt signaling cascades	[43]
SS	Salivary gland epithelial cells	Autoantigenic Ro/SS-A, La/SS-B and Sm RNPs	Present intracellular autoantigens to immune system to induce immune response or tolerance	[51]
SS	EVB-infected B lymphocytes	miR-BART13-3p (exogenous)	Target AQP5 and STIM1, impact activation of a critical Ca2+ entry, impair salivary gland function	[52]
kidney allotransplantation	Tregs generated by dendritic cells transfected with adenovirus-encoding dnIKK2 in vitro	Specifc miRNAs and iNOS enzyme	Inhibit T cell alloreactivity, promote Tregs generation, prolong kidney allograft survival	[53]
Islet transplantation	Human bone marrow-derived MSCs transfected by overexpressed siFas and anti-miR-375 in plasmid	siFas and anti-miR-375 (exogenous)	Silence Fas and miR-375 of human islets, inhibit early apoptosis of transplanted human islets	[54]
Corneal implant	In-growing pig corneal epithelium cells		Generate matrix components, promote corneal regeneration	[55]
Corneal wound healing	Mouse corneal epithelial cells	Thrombospondin-2, latent-transforming growth factor beta-binding protein 1, C-X-C motif chemokine 5, and C-C motif chemokine 2	Trigger keratocyte proliferation, convert keratocyte transformation into myofibroblasts, angiogenesis	[56]
Corneal wound healing	Normal human cornea limbal keratocytes	Small RNAs	Enhance proliferation and wound healing rates of limbal epithelial cells through activating Akt signaling	[57]
Corneal wound healing	Human corneal MSCs		Accelerate corneal epithelial wound healing	[58]
Noninfectious uveitis	ARPE-19		Inhibit T-cell proliferation, regulate human monocyte phenotype and viability	[59]
Autoimmune uveoretinitis	Human bone marrow-derived MSCs		Prevent the onset of EAU by suppressing Th1/Th17 development and inhibiting T cell proliferation	[60]
Autoimmune uveitis	Human umbilical cord-derived MSCs		Exert therapeutic effects on EAU by inhibiting inflammatory cell migration	[61]
AMD	ARPE-19	Complement protein C3	Targets for complement factor H, interact with the complement pathways	[62]
Laser-induced choroidal neovascularization	Mouse retinal astroglial cells	Endostatin, KC/Chemokine (C-X-C motif) ligand 1, macrophage inflammatory protein-1, matrix metalloproteinase-3 and -9, nephroblastoma-overexpressed, pigment endothelium-derived factor, proliferin and tissue inhibitor of metalloproteinases-1	Suppress retinal vascular leakage, reduce choroidal neovascularization	[63]
Atherosclerosis	Mouse bone marrow-derived MSCs	miR-let7 family	Decrease macrophage infiltration via miR-let7/IGF2BP1/PTEN pathway, regulate macrophage polarization via miR-let7/HMGA2/NF-kB pathway	[64]
Cancer	Human bone marrow-derived MSCs	miR-100	Decrease the expression and secretion of VEGF via modulating the mTOR/HIF-1α signaling	[65]
Hyperglycemia-induced retinal inflammation	Human umbilical cord-derived MSCs	miR-126	Suppress the hyperglycemia-induced inflammatory response via downregulating HMGB1 signaling	[66]

This list is limited to studies presented in this review. *Tregs* regulatory T cells, *PD-L1* programmed death-ligand 1, *MSCs* mesenchymal stem cell, *SS* Sjögren’s syndrome, *RNPs* ribonucleoproteins, *EVB* Epstein-Barr virus, *AQP5* aquaporin 5, *STIMI* stromal interacting molecule 1, *iNOS* inducible nitric oxide synthase, *siFas* siRNA against Fas receptor, *ARPE-19* human retinal pigment epithelium cell line, *EAU* experimental autoimmune uveoretinitis, *AMD* age-related macular degeneration, *VEGF* vascular endothelial growth factor, *HMGB1* high-mobility group box 1

## Exosomes in immune-mediated eye diseases

### Sjögren’s syndrome (SS) dry eye

Sjögren’s syndrome (SS), a multisystem autoimmune disease, is characterized by lymphocytic infiltration in salivary and lacrimal glands (LGs) and the presence of various autoantibodies (such as anti-Ro(SS-A) or anti-La(SS-B)), resulting in oral and ocular dryness [67, 68]. This condition leads to one of the most severe subtypes of dry eye diseases [20]. Activation of both innate and adaptive immune pathways, such as interferon (IFN) signatures, B cell activating factor (BAFF)/BAFF receptor axis, and NF-kB signaling, contributes to the pathogenesis of SS [69, 70].

Salivary gland epithelial cells (SGECs) in SS play active roles in the autoimmune and inflammatory responses by virtue of the constitutive or inducible expression of diverse immunoactive factors, such as BAFF, several Toll-like receptors (TLRs), and autoantigenic ribonucleoproteins (RNPs) [71, 72]. Lymphocytic infiltrates consisting primarily of CD4^+^ T cells and B cells occur proximally to and frequently invade epithelial cells [73, 74], suggesting the interaction between epithelial and immune cells. One previous study demonstrated that the autoantigenic Ro/SS-A, La/SS-B, and Sm RNPs were present in exosomes which were released continuously by SGECs, indicating that intracellular autoantigens were transferred to autoreactive lymphocytes via RNP-containing exosomes. However, this release is not restricted to SS-derived cells [51]. Besides, as EBV typically infects B cells, one study proposed that EBV-miRBART13-3p could be transferred via exosomes from B cells to SGECs. This functional miRNA targeted aquaporin 5 (AQP5) and stromal interacting molecule 1 (STIM1), which could significantly impact salivary secretion. However, the authors did not mention the effect on the function of LGs [52].

The LGs are primarily responsible for the aqueous layer of the tear film. LG dysfunction is mainly due to the infiltration of immune cells [75]. Our research team has verified that MSC administration efficiently alleviated induced autoimmune dacryoadenitis in rabbit models, which closely mimic human SS [76]. It is noted that MSC-Exos mediate the immunosuppressive effects of their parent cells and are deemed as promising surrogates for MSC-based therapy [33]. Ongoing studies in our laboratory recently demonstrated that subconjunctivally administered MSC-Exos efficiently improved clinical evaluations and diminished the inflammation in lacrimal glands of diseased rabbits, compared with those treated with saline. The therapeutic effects may partially be ascribed to their modulatory effects on lacrimal macrophage polarization and enhancement of Treg and Th2 responses via targeting NF-kB signaling. Therefore, MSC-Exos presumably provide a very promising cell-free therapy for SS dry eye. In addition, the role of exosomes in interactions between lymphocytes and LG epithelial cells remains unexplored, calling for extensive research.

### Corneal allograft rejection

Corneal transplantation is the most prevalently performed type of tissue grafting globally. To enhance corneal graft survival, considerable efforts have been devoted to building effective strategies [77]. Although cornea as an avascular transparent tissue enjoys the relative privilege of immunity, the major cause of corneal graft failure reported is allogeneic rejection, which is ascribed to the adaptive immune response initiated through recognition of donor MHC antigens by recipient T cells after transplantation [78, 79]. EVs, including exosomes, released by donor cells are partly responsible for this type of allorecognition [80]. Howbeit, they also contribute to allograft tolerance under certain circumstances. It has been reported that EVs from a specific population of CD4^+^CD25^−^ Tregs generated in vitro could prolong kidney allograft survival, which was mediated by their unique cargo, specific miRNAs, and inducible nitric oxide synthase (iNOS) enzyme [53]. Moreover, MSC-Exos loaded with specific small RNAs successfully improved islet transplantation [54]. These encouraging results suggest that exosomes from specific immunosuppressive cell populations serve as a potentially effective tool to promote immune tolerance in graft survivals such as corneal graft.

For decades, severe global shortfall of donated human corneas has been an ongoing challenge that should not be ignored [81]. To address this, new biomaterials, such as collagen gels, synthetic polymers, and tissue-engineered scaffolds, have been developed to repair, regenerate, or replace the damaged cornea [82]. Jangamreddy et al. found that one kind of peptide analogs as alternatives to collagen promoted regeneration of corneal tissue by stimulating in-growing corneal epithelium cells to secrete EVs for generating matrix components [55]. During corneal wound healing, mouse corneal epithelial cell-derived exosomes induced fibroblast proliferation and transformation of keratocytes to myofibroblasts, mediating intercellular communication between the corneal epithelium and stroma [56]. Besides, exosomes derived from normal human corneal limbal keratocytes were found to greatly enhance proliferation and wound healing rates of primary limbal epithelial cells, likely via activating Akt signaling [57]. One recent study revealed that human corneal MSC-Exos were capable of accelerating corneal epithelial wound healing [58]. Together, the available results indicate that exosomes are vital biological mediators of regeneration [83] and provide new insights into the therapeutic strategies for corneal injury and transplant rejection.

### Autoimmune uveitis

Autoimmune uveitis, an inflammation of the uvea (iris, ciliary body, and choroid tissue) and even adjacent tissues (vitreous humor, optic nerve and retina), can occur either alone or secondary to systemic syndrome [84]. The autoimmune causes are mainly due to inappropriate immune responses mediated by pathogenic T cells [85]. Pathogenic Th17 cells and their related inflammatory cytokines coordinately act as potent inducers of tissue inflammation [86, 87]. Innate immune cells such as DCs, monocytes/macrophages, γδT cells, natural killer (NK) cells, and NKT cells also actively participate in shaping the effector T cell responses in autoimmune uveitis [88, 89].

During the inflammatory processes, particularly in posterior uveitis, retinal pigment epithelium (RPE) cells may get damaged [90]. RPE cells have been revealed to have immunosuppressive properties, including induction of Tregs and inhibition of Th17 and Th22 cell differentiation [91]. Knickelbein et al. reported that exosomes released by both resting and cytokine-stimulated RPE cells suppressed the proliferation of T lymphocytes isolated from the peripheral blood of noninfectious uveitis patients, and these nanosized vesicles could also regulate human monocyte phenotype and viability [59]. The above results indicate that exosome secretion may be a crucial mechanism for RPE cells to perform their immunoregulatory effects. Further understanding of exosomes from RPE cells may reveal novel vistas for therapy of uveitis.

Interestingly, Shigemoto-Kuroda and colleagues found that human bone marrow-derived MSC-Exos could effectively ameliorate experimental autoimmune uveoretinitis (EAU). The mixed lymphocyte reaction assay indicated that these MSC-Exos performed a significant inhibitory effect on the T cell proliferation and Th1 and Th17 development [60]. However, in another experimental study focused on EAU, human umbilical cord-derived MSC-Exos (hUC-MSC-Exos) failed to suppress the proliferation of conA-stimulated T cells, but effectively inhibited inflammatory cell migration [61]. In vitro results from our group showed that hUC-MSC-Exos had only a slight suppressive effect on interphotoreceptor retinoid-binding protein (IRBP)-specific Th17 responses, while they significantly inhibited DC-driven Th17 responses through the modulation of DC-derived Th17-polarizing cytokines IL-1β, IL-6, and IL-23. The discrepancies of these results may be due to the high heterogeneity of exosomes and distinct assay systems applied in the studies. It thus appears that MSC-Exos have therapeutic potential for autoimmune uveitis, but the specific mechanism related to their anti-inflammatory and immunomodulatory effects warrants further investigations.

### Age-related macular degeneration (AMD)

Age-related macular degeneration (AMD), a complex multifactorial degenerative disease, is a leading cause of blindness among the elderly in developed countries [92]. Two clinical phenotypes of AMD exist: early non-exudative (dry-type) and late exudative (wet-type). The dry-type AMD is characterized by yellowish drusen (accumulation of extracellular deposits) and geographic atrophy, whereas the wet-type involves choroidal neovascularization (CNV) [93].

Gradually, it has been realized that pathological processes in AMD which had once been considered to be purely degenerative also implicate immune and inflammatory elements [21]. The complement system, a major arm of the innate immunity, has been recognized as a key component in AMD pathogenesis [94]. Reportedly, reduced membrane complement regulators in RPE cells contributed to RPE damage in AMD, and the decreased levels were partially explained by their release in apoptotic particles and exosomes [95]. Single nucleotide polymorphisms (SNPs) in complement factor H (CFH) gene have been identified to be linked with an increased risk of developing AMD [96, 97]. The CFH gene encodes protein factor H (FH) which functions as a regulator of the complement pathway [96]. Taylor et al. recently proposed that haploinsufficiency of factor H-like 1 (FHL-1), a variant of FH serving as a major complement regulator in Bruch’s membrane, may be an important mechanism driving the development of early-onset macular drusen in the vast majority of AMD cases [98]. Also, loss of complement protein C3 functionality contributes to the pathogenesis of AMD [99]. Dysfunction of CFH may cause C3-coated exosomes from RPE cells to become attacked by the invading leukocytes in the aged retina, and this might cause destabilization of exosome membranes and then result in the release of intracellular proteins, contributing to the formation of drusen [62]. These imply that RPE cell-derived exosomes are in part responsible for complement-driven innate immune responses in AMD.

In exudative AMD, especially in the CNV membranes, macrophages are the major populations of infiltrating inflammatory cells [100]. A pathological switch of macrophage polarization may be implicated in the development of CNV [101]. Retinal astrocyte-derived exosomes were confirmed to target both macrophages and vascular endothelial cells and perform significant inhibitory effects on laser-induced retinal vessel leakage and CNV of mouse models [63]. Besides, vascular endothelial growth factor (VEGF) has been identified as a critical inducer of pathologic neovascularization [102]. It is known that MSC-Exos are capable of regulating macrophage polarization [64] and downregulating VEGF expression [65]. Thereout, it can be speculated that MSC-Exos have the potential to control aberrant neovascularization in exudative AMD.

## Exosome biomarkers for eye diseases

Exosomes and other EVs, particularly their cargoes, have been increasingly recognized as ideal low-invasive biomarkers in detecting, monitoring, and prognosticating diseases in recent years [103]. Especially in cancer screening, thermophoretic aptasensor has been developed to profile surface proteins of serum EVs for early cancer detection and classification [104]. Exosomes are abundant in tear fluids [105], aqueous humor (AH) [106], vitreous humor (VH) [107], and blood [108], all of which are important body fluids associated with ocular health and disease. Though it is less developed, theoretically, the identification and characterization of exosome-specific biomarkers in eye diseases have a great significance. For example, exosomes and their miRNA payload or proteomic profiling in AH may be used as novel diagnostic biomarkers for patients with glaucoma and neovascular AMD [106, 109]. Proteomic findings of RPE-derived exosomes may also offer diagnostic indicators for retinal disease [110]. Furthermore, Ragusa and colleagues showed that miR-146a was significantly upregulated in the VH exosomes of uveal melanoma patients with respect to controls, and the upregulation was also detected in serum exosomes of the same patients. Based on this, exosome-derived miR-146a might be deemed as a potential marker of uveal melanoma [107]. Overall, with the recent progress in exosome-specific isolation techniques and identification methods for their protein and nucleic acid contents, the research of exosome biomarkers for eye diseases appears to have sufficiently hopeful prospects.

## Exosomes as drug delivery vesicles

The conventional route of treatment for eye disease, especially involving the anterior segment, is topical instillation of eye drops, which is accompanied with limitations such as the need for frequent administration and low bioavailability. During recent years, various synthetic drug vehicles have been developed for encasing existing drugs to enhance the therapeutic effect [111]. However, troubling issues including their immunotoxicity [112] and quick clearance by the mononuclear phagocyte system (MPS) or the reticuloendothelial system (RES) [113] still exist. Fortunately, exosomes, regarded as natural nanocarriers, have plenty of the highly desired qualities that drug delivery vehicles should have. These small vesicles are capable of penetrating the blood-brain barrier (BBB), delivering their cargoes across cell membranes and targeting specific cell types after artificial modifications [114]. Collectively, exosomes have been shown to serve as possible nanocarriers for functional RNA strands (mRNA, miRNA, siRNA, and lncRNA), DNA molecules, peptides, or synthetic drugs [115, 116]. For instance, exosomes from adeno-associated virus type 2 (AAV-2)-producing 293 T cells showed higher efficiency in retinal transduction than conventional AAV-2 after intravitreal injection and were regarded as robust tools for intravitreal gene transfer into the retina [117]. Besides, MSC-Exos loaded with exogenous miRNA-126 were reported to alleviate hyperglycemia-induced retinal inflammation via suppressing the high-mobility group box 1 (HMGB1) signal pathway [66]. Moreover, chemotherapeutic drug-loaded exosomes showed higher efficacy and better bioavailability compared to free drug [118, 119], which sheds new light on ocular pharmacotherapeutics. So far, there has been sparse research focused on the latent role of loading exosomes with exogenous functional cargoes in eye diseases. Therefore, significant endeavors are needed to develop such therapies in ophthalmology.

## Conclusions

**Fig. 2:**
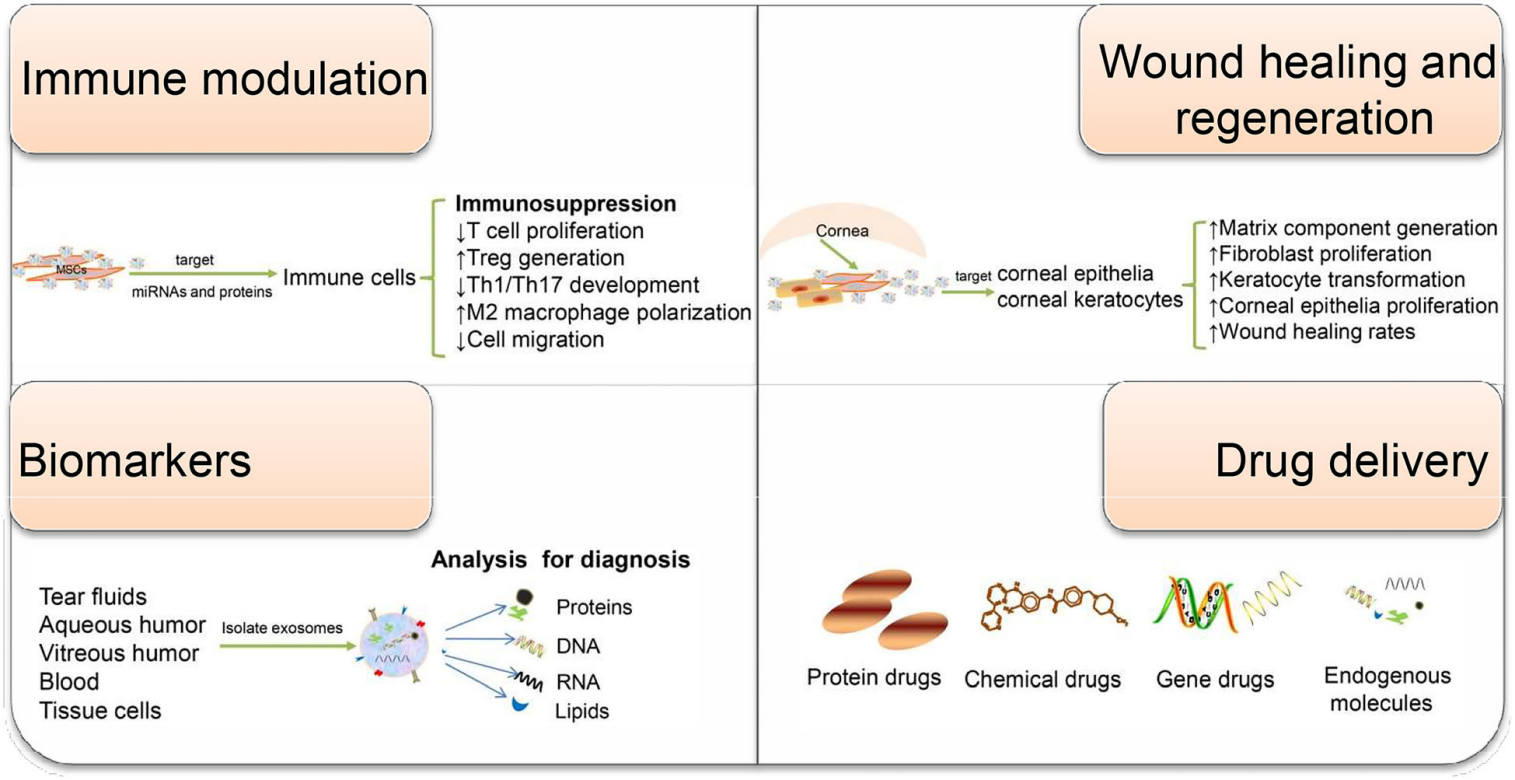
Schematic of the potential application of exosomes in immune-mediated eye diseases. Exosomes have been involved in a broad variety of physiological and pathophysiological events. Depending on their origin and exposure context, they exert different functions including intercellular communication, immune modulation, wound healing, and regeneration. MSC-Exos have been found to regulate the activity of intraocular immune cells. Corneal epithelial cell-derived exosomes are capable of promoting matrix component generation, and corneal limbal keratocyte-derived exosomes can accelerate corneal wound healing. Moreover, exosomal miRNA payload or proteomic profiling can reflect the disease state and have the potential to serve as eye disease-specific biomarkers. Owing to their highly desired drug carrier attributes, exosomes are increasingly considered as ideal drug delivery systems. Together, exosome-based therapy or diagnosis holds great potential for clinical application in ophthalmology

Taken together, the extensive implication of exosomes in regulating various aspects of the immunity makes exosomes attractive diagnostic and therapeutic candidates for immune-mediated eye diseases (Fig. 2). Because of their multiple functions, elucidating the contents of exosomes and understanding how each of them function are necessary. Additionally, for successful translation into clinical therapies, novel and advanced technology is urgently needed to obtain mass highly purified exosomes with stable functional efficacy. Exosome research in the eye is still a relatively young field, awaiting more extensive investigations into the precise biological mechanisms and clinical potential of exosomes in ocular diseases.

## Data Availability

Not applicable.

## Abbreviations

AAV-2: Adeno-associated virus type 2
AH: Aqueous humor
AMD: Age-related macular degeneration
AQP5: Aquaporin 5
BAFF: B cell activating factor
BBB: Blood-brain barrier
CFH: Complement factor H
CNV: Choroidal neovascularization
DCs: Dendritic cells
EAU: Experimental autoimmune uveoretinitis
EBV: Epstein-Barr virus
ESCRT: Endosomal sorting complex required for transport
EVs: Extracellular vesicles
FH: Factor H
FHL-1: Factor H like 1
HMGB1: High-mobility group box 1
hUC-MSC-Exos: Human umbilical cord-derived MSC-Exos
IFN: Interferon
ILV: Intraluminal vesicles
iNOS: Inducible nitric oxide synthase
IRBP: Interphotoreceptor retinoid-binding protein
LGs: Lacrimal glands
MHC: Major histocompatibility complex
MPS: Mononuclear phagocyte system
MSC-Exos: Mesenchymal stem cells-derived exosomes
MSCs: Mesenchymal stem cells
MVBs: Multivesicular bodies
NK: Natural killer
PD-L1: Programmed death-ligand 1
RES: Reticuloendothelial system
RNPs: Ribonucleoproteins
RPE: Retinal pigment epithelium
SGECs: Salivary gland epithelial cells
SNARE: Soluble *N*-ethylmaleimide-sensitive fusion attachment protein receptor
SNPs: Single nucleotide polymorphisms
SS: Sjögren’s syndrome
STIM1: Stromal interacting molecule 1
TLRs: Toll-like receptors
Tregs: Regulatory T cells
TSG101: Tumor susceptibility gene 101
VEGF: Vascular endothelial growth factor
VH: Vitreous humor
